# Fishing for (in)continence: long-term follow-up of women with OASIS–still a taboo

**DOI:** 10.1007/s00404-020-05878-8

**Published:** 2020-12-01

**Authors:** Sabine Schütze, Benedikt Hohlfeld, Thomas W. P. Friedl, Stephanie Otto, Katrina Kraft, Katharina Hancke, Beate Hüner, Wolfgang Janni, Miriam Deniz

**Affiliations:** 1 Department of Gynaecology and Obstetrics, Ulm University Hospital, Ulm, Germany; 2 Kbo-Lech-Mangfall Clinic Garmisch-Partenkirchen, Garmisch-Partenkirchen, Germany; 3 Comprehensive Cancer Center Ulm (CCCU), Ulm University Hospital, Ulm, Germany; 4 München Klinik Harlaching, Munich, Germany

**Keywords:** OASIS, Long-term outcome, Pelvic floor function, Flatus incontinence, Urinary incontinence, Quality of life

## Abstract

**Purpose:**

Obstetric anal sphincter injuries (OASIS) increase the risk for pelvic floor dysfunctions. The goal of this study was to examine the long-term outcomes after OASIS on pelvic floor functions and quality of life.

**Material and methods:**

Between 2005 and 2013, 424 women had an OASIS at the Women University Hospital Ulm. Out of these 71 women completed the German pelvic floor questionnaire, which includes questions regarding prolapse symptoms as well as bladder, bowel and sexual function. In addition, 64 women were physically examined, including a speculum examination to evaluate the degree of prolapse, a cough test to evaluate urinary stress incontinence (SI) and an evaluation of both pelvic floor sphincter (modified Oxford score) and anal sphincter contraction.

**Results:**

A high rate of pelvic floor disorders after OASIS was found, as 74.6% of women reported SI, 64.8% flatus incontinence and 18.3% stool incontinence, respectively. However, only few women stated a substantial negative impact on quality of life. The clinical examination showed that a positive cough test, a weak anal sphincter tone and a diagnosed prolapse correlated with the results of the self-reported questionnaire.

**Conclusion:**

On one hand, OASIS has an influence on pelvic floor function going along with lots of complaints, while on the other hand, it still seems to be a taboo topic, as none of the participants spoke about the complaints after OASIS with a doctor. Therefore, the gynecologist should actively address these issues and offer therapy options for the women with persisting problems.

## Introduction

Many women suffer from pelvic floor disorders following pregnancy and delivery, which may occur immediately after birth or many years later. The functional disorders include urinary stress incontinence (SI), overactive bladder, anal incontinence and complaints related to prolapse. In addition, sexuality is often negatively affected after delivery [2, 4, 33]. Nygaard et al. described in a retrospective study in US women a high prevalence of symptomatic pelvic floor disorders. The proportion of women reporting disorders increased significantly with age and parity, and was higher in overweight and obese as compared to normal weight women [20]. Due to the rising birth rate as well as a rising number of obese women in Germany, the prevalence of pelvic floor disorders is likely to increase.

It is known that up to 33% suffer of urinary incontinence and 10% from stool incontinence after delivery [11], and up to two thirds of women develop a pelvic floor prolapse over time [5, 32]. All of these pelvic floor dysfunctions can have a serious impact on the quality of life and should therefore be detected, treated and if–possible–prevented.

Additional risk factors for the pelvic floor following vaginal birth include vaginal operative delivery (forceps or vacuum), fetal macrosomia and delivery with occiput posterior position [10, 18]. The influence of obstetric anal sphincter injuries (OASIS) on the long-term prevalence of urinary incontinence, pelvic floor prolapse or sexuality has barely been studied, and the results of the few existing studies are contradictory. Some studies have shown that the highest risk for pelvic floor dysfunction, including a high risk for flatus or stool incontinence, is a delivery with an OASIS [19, 31]. This is in contrast to the study of Nygaard et al. They carried out a 30-year retrospective study with the focus on anal incontinence after OASIS with two control groups (episiotomy without extension to the anal sphincter; cesarean section). Regardless of the type of delivery, anal incontinence occurred in a surprisingly large number of the women [22]. However, the number of included women in that study was very small. Therefore, more studies examining the long-term outcome after OASIS are necessary. The goal of our study was to retrospectively analyze quality of life and pelvic floor function of women in long-term follow-up after an OASIS event during vaginal birth and to compare the results of a clinical examination with self-reported pelvic floor function disorders.

## Materials and methods

### Study population

**Fig. 1:**
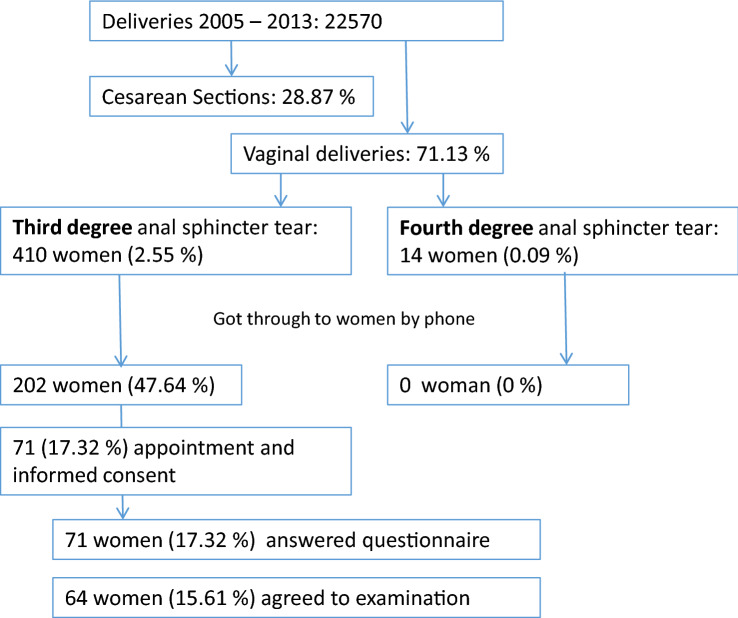
Study population

We screened all patients who experienced a perineal injury grade 3 or 4 during a vaginal delivery at the Department of Gynecology and Obstetrics of the University Hospital Ulm, Germany, occurring at least 2 years before the beginning of data collection for the study in 2015. Between 2005 and 2013, there were 16,055 vaginal deliveries with 424 documented cases of OASIS (perineal injury grade 3 or 4). Exclusion criteria for this study was a pelvic organ prolapse or incontinence surgery in the past. Every case was diagnosed and treated by an attending physician. In this hospital, the mediolateral episiotomy is used when there is a risk of fetal compromise, while median episiotomy is not practiced. According to the German AWMF Guideline women usually got an appointment in a specialized consultation hour 8 to 12 weeks after suffering from a perineal tear. All 424 women were contacted via telephone, invited to participate in the study and received a written invitation. 71 women finally participated in our study; all of them completed the questionnaire and 64 women agreed to additional examination (see Fig. 1). The study protocol was submitted to and approved by the ethics committee of the University Ulm (No. 339/15), and written informed consent was obtained from all participants.

### Questionnaire, interview and data collection

The validated German pelvic floor questionnaire (Deutscher Beckenbodenfragebogen) consists of 42 questions, evaluating quality of life and encompassing the four domains of bladder function (15 questions), bowel function (12 questions), prolapse symptoms (5 questions), and sexual function (10 questions) [1]. Each question is individually scored. The four domains were scaled from 1 (no dysfunction, favorable) to 10 (maximal dysfunction, unfavorable), and the domain scores were then summed to obtain a total pelvic floor dysfunction score ranging from 0 (no limitation/dysfunction) to 40 (all four domains maximally dysfunctional). Please note that the two response categories “moderately” and “greatly” for question 32 (“How much of a bother is the prolapse to you?”) were combined to one category “moderately/greatly”; similarly, the two response categories “minimal” and “none” for question 36 (“During intercourse vaginal sensation is…”) were combined to one category “minimal/none”. In addition, the patients were interviewed and the following information was collected: parity, body mass index (BMI), prolapse complaints, urinary incontinence, stool incontinence and sexual activity. From our obstetrical database, we collected the following information: weight at delivery, delivery mode (spontaneous, vacuum extraction,), episiotomy, epidural anesthesia, length of the first (latent) stage of labor, length of the second (active) stage of labor, fetal position (occipital posterior, occipital anterior, breech) and maternal birth position.

### Clinical examination

The physical examination included a speculum examination to evaluate degree of the prolapse with the help of the pelvic organ prolapse quantification system (POP-Q [24]), which quantifies the descent of pelvic organs into the vagina and comprises five stages from stage 0 (no prolapse) to stage 4 (total prolapse). A cough test (positive or negative) was used to evaluate SI. Pelvic floor contraction was evaluated using the Oxford Score [13], which rates pelvic floor muscle contraction on a scale from 0 to 5. We used a modified score ranging from 1 to 5 (1 = no contraction, 2 = weak muscle contraction, 3 = moderate muscle contraction, 4 = good muscle contraction, 5 = strong muscle contraction). Anal sphincter contraction was categorized as strong (i.e., normal) or weak (i.e., reduced).

### Statistical analysis

Ordinal and categorical variables are summarized and described using absolute and relative frequencies, while continuous variables are described using medians and ranges.

Associations among domain scores of the questionnaire as well as correlations between the metric variables maternal age at the time of the birth with an OASIS (years), maternal BMI at the time of the birth with an OASIS (kg/m^2^), fetal birth weight (g), fetal head circumference (cm), time between birth with OASIS and completion of questionnaire (months), or duration of the first and second stage of labor (min) and total score as well as the four domain scores of the pelvic floor questionnaire were analyzed based on the Spearman rank correlation coefficient *r*_s_. Associations between the ordinal-scaled modified Oxford score and questionnaire scores were also evaluated using the Spearman rank correlation test. Associations between the categorical variables maternal birth position (supine vs. non-supine), epidural anesthesia (yes vs. no), delivery mode (spontaneous vs. vacuum-extraction), episiotomy (yes vs. no), POP-Q score (in this study only scores 0, 1, or 2), anal sphincter contraction (strong vs. weak), or cough test (positive vs. negative) and questionnaire scores were evaluated based on group comparisons of scores between categories using the Mann–Whitney *U* test (two categories) or the Kruskal Wallis *H* test (more than two categories).

For the analyses evaluating associations between the results of the physical examinations (POP-Q score, modified Oxford score, anal sphincter contraction and cough test) and questionnaire scores, only women were included who had answered all questions used to calculate the scores.

All statistical tests were two-sided, and *p* values below 0.05 were regarded as significant. As this is an explorative study, no adjustment of significance level for multiple testing was performed. All statistical analyses were conducted using the SPSS statistical software package, version 25 (IBM, New York, NY, USA).

## Results

### Maternal, obstetrical and fetal characteristics

71 women participated in the study, whereas none of them had a perineal injury grade 4. In the delivery protocol only in 21 cases the OASIS was further subdivided into grade A (14 cases), grade B (5 cases) and grade C (3 cases).

Median age of the 71 participating women at the time of the OASIS-associated delivery was 32 years, and in 50 (70.4%) women the OASIS-associated delivery was the first delivery. Median time between the birth with OASIS and clinical examination/completion of the questionnaire for this study was 67 months (about 5.5 years) with a range from 2.4 to 11.1 years. More than 50% of the women gave birth in supine position, and almost 80% had a spontaneous mode of delivery. More detailed and additional baseline maternal, obstetrical and fetal characteristics for the 71 cases of vaginal births with an obstetric anal sphincter injury (OASIS) included in this study are shown in Table 1. In the past, 40 out of the 71 women had kept their appointment in the specialized consultation hour 8 to 12 weeks after delivery.

**Table 1:** Baseline maternal, obstetrical and fetal characteristics of vaginal births with an obstetric anal sphincter injury (OASIS) (*n* = 71)

Variable	
Maternal age at birth (years)	
Median	32
Range	19–42
Number of missing values	0
Maternal body mass index at birth (kg/m^2^)	
Median	23.8
Range	18.3–37.7
Number of missing values	2
Time between birth with OASIS and clinical examination/completion of questionnaire (months)	
Median	67
Range	29–133
Number of missing values	0
Parity	
1	17 (23.9%)
2	42 (59.2%)
3 or higher	12 (16.9%)
Missing	0 (0.0%)
Delivery with OASIS	
First	50 (70.4%)
Second	18 (25.4%)
Third	3 (4.2%)
Missing	0 (0.0%)
Duration of the first stage of labor (latent phase, shortening and opening; min)	
Median	390
Range	90–1375
Number of missing values	18
Duration of the second stage of labor (delivery; min)	
Median	75
Range	6–319
Number of missing values	2
Maternal birth position	
Supine	39 (54.9%)
Lateral	6 (8.5%)
Upright	3 (4.2%)
Missing	23 (32.4%)
Fetal birth position	
Occiput anterior	62 (87.3%)
Occiput posterior	7 (9.9%)
Breech	1 (1.4%)
Missing	1 (1.4%)
Mode of delivery	
Spontaneous	56 (78.9%)
Vacuum extraction	15 (21.1%)
Missing	0 (0.0%)
Epidural anesthesia	
Yes	43 (60.6%)
No	28 (39.4%)
Missing	0 (0.0%)
Episiotomy	
Yes	24 (33.8%)
No	46 (64.8%)
Missing	1 (1.4%)
Fetal weight at birth (g)	
Median	3350
Range	2370–4940
Number of missing values	0
Fetal head circumference at birth (cm)	
Median	35
Range	32–38
Number of missing values	0

### Clinical examination and pelvic floor assessment

The results of the physical examination are presented in Table 2. Overall, 35 (49.3%) women showed some degree of prolapse and the highest POP-Q stage found in our study was stage 2 with 13 (18.3%) women being affected. No contraction of the pelvic floor (modified Oxford score) was found in 14 (19.7%) women. A weak contraction of the anal sphincter during the examination was found in 29 (40.8%) of the women. SI as defined by urine loss at the cough test was found in 44 (62.0%) of the participants. Pelvic floor contractibility (modified Oxford score) was significantly associated with anal sphincter contraction (*p* = 0.008), but not with POP-Q stage (*p* = 0. 31) or cough test result (*p* = 0.81).

**Table 2:** Results of clinical examinations regarding degree of prolapse (Pelvic Organ Prolapse Quantification Score POP-Q), Pelvic Floor Sphincter Contraction (modified Oxford Score from 1 to 5), contraction of the anal sphincter and stress urinary incontinence (assessed using a cough test) in women with an obstetric anal sphincter injury (OASIS) during vaginal birth (*n* = 71)

Variable	
POP-Q Score	
0	29 (40.8%)
1	22 (31.0%)
2	13 (18.3%)
3	0 (0.0%)
4	0 (0.0%)
Missing	7 (9.9%)
Modified Oxford Score	
1	14 (19.7%)
2	12 (16.9%)
3	15 (21.1%)
4	9 (12.7%)
5	16 (22.5%)
Missing	5 (7.0%)
Anal sphincter contraction	
Strong	33 (46.5%)
Weak	29 (40.8%)
Missing	9 (12.7%)
Stress urinary incontinence	
Yes	44 (62.0%)
No	23 (32.4%)
Missing	4 (5.6%)

### German pelvic floor questionnaire

#### Total score

Overall, 46 women answered all questions. The median total score was 3.6 (range 0.4–11.7). 26 (56.5%) women had a total score less than 4.0. The bladder function, bowel function and sexual function domain scores were significantly correlated with each other (Spearman rank correlation, all *r*_s_ > 0.35; all *p* < 0.02), while there was no significant correlation of the prolapse symptoms domain score with any of the other three domain scores (all *p* > 0.4).

#### Bladder function

Stress urinary incontinence (SI) occurs when the intraabdominal pressure increases, as sneezing, laughing, coughing, which leads to a loss of urine. In our cohort, 53 (74.6%) of the women reported urine loss due to stress incontinence, and 7 (9.9%) complained about loss of urine during activity every day. Urinary pads were needed in 21 (29.6%) of the women (prophylactically in 14.1%; during exercise 4.2%; daily in 11.3%). Almost one third (19 women, 26.7%) stated a reduction on the quality of life due to the SI, with 3 (4.2%) women reporting a drastic impact of SI on quality of life. An overactive bladder (OAB), defined as a sudden, uncontrolled need or urge to urinate, is often accompanied with the need to pass urine many times during the day and night time. It may lead to the loss of urine (urge incontinence). The symptoms of an OAB were experienced sometimes (less than once a week) in 36 (50.7%) women, several times a week in four (5.6%) women and daily in four (5.6%), while 3 (4.2%) women suffered from urge incontinence, i.e., urine leak when they rush/hurry to the toilet. Nocturia, defined as a urinary frequency of more than once a night, was reported by eight (11.2%) of the women, and five (7.0%) of the women complained of nocturnal enuresis. To avoid SI as well as the symptoms of an OAB, eight (11.2%) of the women reduced their daily fluid intake. The median score of the bladder function domain for women that answered all questions of this domain (*n* = 63) was 0.9 (range 0–4.2).

#### Bowel function

**Fig. 2:**
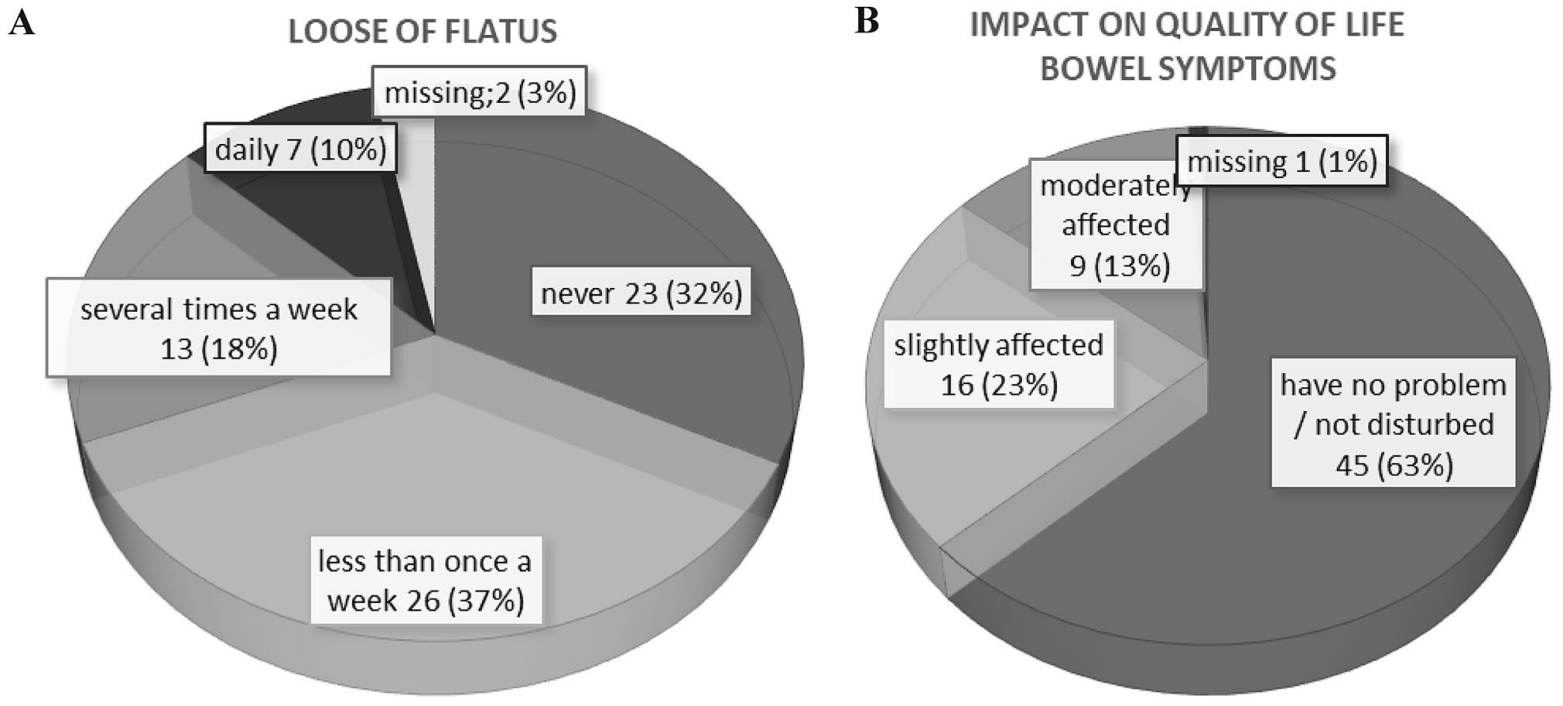
Bowel functions of women after OASIS. **a** Response frequencies to the question regarding flatus incontinence (“Do you accidentally lose flatus?”). **b** Response frequencies to the question “How much are you disturbed by your bowel problems?”)

Flatus incontinence was reported by 46 (64.8%) women in our collective, with 20 (28.2%) women suffering from flatus incontinence several times a week or even daily (Fig. 2). Occasional or rare incontinence of soft stool and formed stool was reported by 13 (18.3%) and 1 (1.4%) of the women, respectively. A moderate decrease in the quality of life as a result of bowel dysfunction was reported by nine (12.7%) women (Fig. 2). The median score of the bowel function domain for women who answered all questions of this domain (*n* = 65) was 1.5 (range 0–4.7).

#### Prolapse symptoms

Complaints because of prolapse (pain or pressure in genital region) were rarely reported in this study, and only ten (14.1%) women stated that they were bothered by prolapse. The median score for the prolapse symptoms domain for women who answered all questions of this domain (*n* = 65) was 0 (range 0–6).

#### Sexual function

Regular sexual activity was reported by 41 (57.7%) women, while 25 (35.2%) women and 1 (1.4%) women indicated rare or no sexual activity, respectively. The reasons for rare or no sexual activity were stated by only six of the women and included little interest, difficulty or pain during intercourse and other reason. Negative impacts on sexual function reported were decrease of lubrication (12 women, 16.9%), decrease of vaginal sensation during intercourse (9 women, 12.7%), occasional or frequent pain during intercourse (38 women, 53.5%), the feeling of a wide vagina (21 women, 29.6%), the feeling of a narrow vagina (13 women, 18.3%) and the loose of urine during intercourse (5 women, 7.0%). A slight reduction in quality of life due to these limitations in sexual functioning was reported by 16 (22.5%) women, while 6 (8.5%) women stated a moderate or strong negative impact on quality of life. The median score for the domain sexual function for women who answered all questions of this domain (*n* = 50) was 1.2 (range 0–4.3).

### Associations between maternal, newborn and fetal characteristics of vaginal births with OASIS and pelvic floor questionnaire scores

There were no significant correlations between the variables maternal age at the time of the birth with an OASIS (years), maternal BMI at the time of the birth with an OASIS (kg/m^2^), newborn birth weight (g), newborn head circumference (cm), time between birth with OASIS and completion of questionnaire (months), or duration of the second stage of labor (min) and any of the four domain scores or the total score of the pelvic floor questionnaire (Spearman rank correlation, all *p* > 0.17). Duration of the first stage of labor (min) was significantly correlated with the prolapse symptoms domain score (*r*_s_ = 0.302, *p* = 0.039), but not with any of the other domain scores or the total score of the pelvic floor questionnaire (all *p* > 0.18). Supine delivery, when compared to non-supine maternal delivery positions, was associated with a higher bladder function domain score (*p* = 0.008), a higher bowel function domain score (*p* = 0.047), a higher sexual function domain score (*p* = 0.031), and a higher total score of the pelvic floor questionnaire (*p* < 0.001), but not with a higher prolapse symptoms domain score (*p* = 0.21). Epidural anesthesia during delivery was associated with a higher sexual function domain score (*p* = 0.003) and a higher total score (*p* = 0.035), but not with a higher bladder function domain score (*p* = 0.22), a higher bowel function domain score (*p* = 0.10) or a higher prolapse symptoms domain score (*p* = 0.86). A vacuum delivery was significantly associated with a higher bowel function domain score (*p* = 0.031), but not with a higher bladder function domain score (*p* = 0.08), a higher prolapse symptoms domain score (*p* = 0.45). a higher sexual function domain score (*p* = 0.18), or a higher total score of the pelvic floor questionnaire (*p* = 0.29). Episiotomy (yes vs. no) was not significantly associated with higher domain scores or a higher total questionnaire score (all *p* > 0.05).

In the documents from the appointment after 8 to 12 weeks, 11 out of 40 women stated to have slight problems with flatus incontinence or rectal tenesmus. Interestingly, there was no correlation of these symptoms 3 months after delivery and a resulting higher stool score in our study.

### Associations between physical examination results and pelvic floor questionnaire scores

**Fig. 3:**
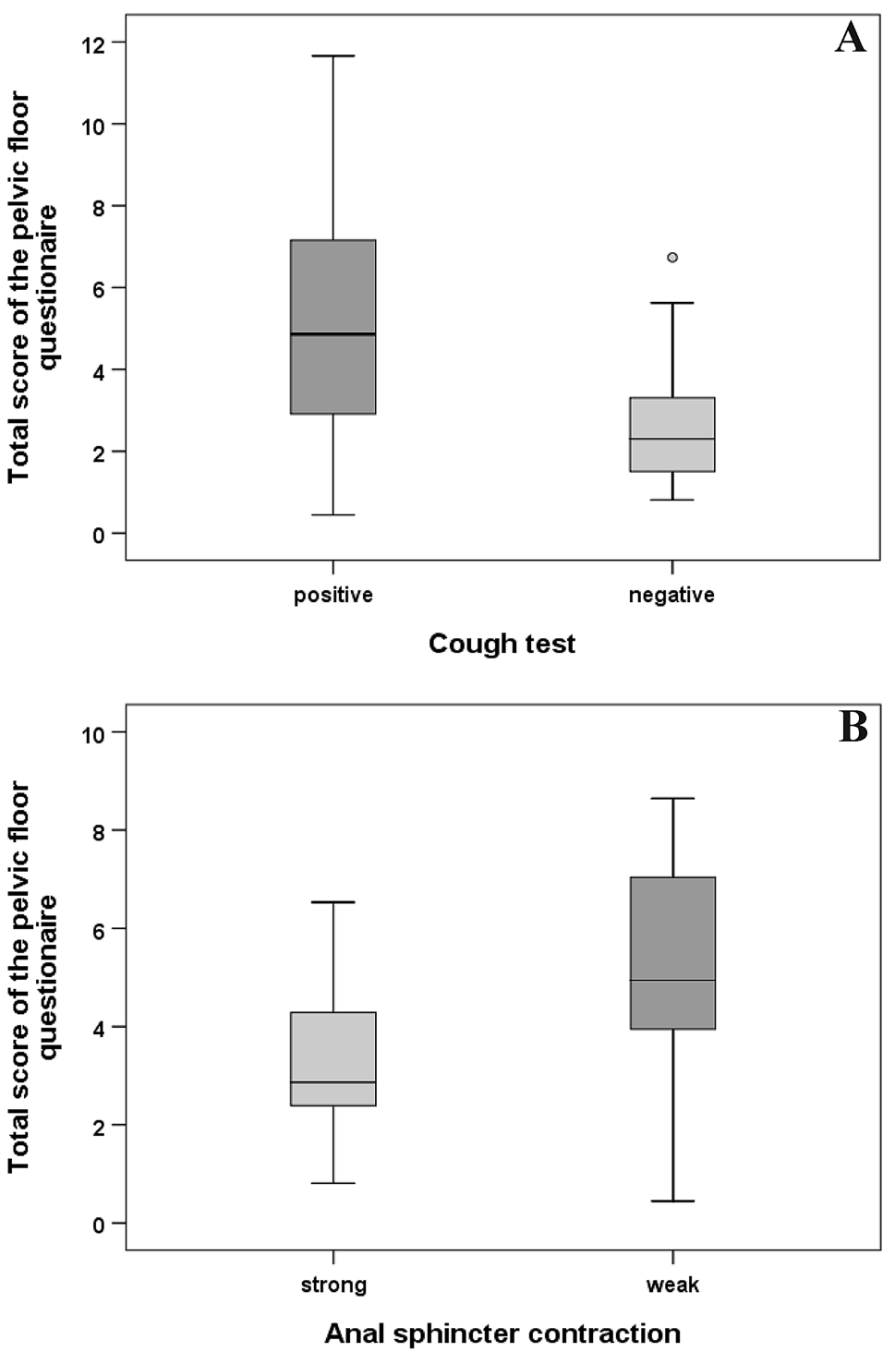
Comparison of total scores of the German pelvic floor questionnaire between women with a positive or negative cough test result (**a**), and between women with weak (reduced) or strong (normal) anal sphincter contraction (**b**). Please note that higher questionnaire scores indicate a higher degree of dysfunction

Women with a positive cough test (i.e., urine stress incontinence) had a significantly higher total questionnaire score (*p* = 0.008, Fig. 3a), bladder function domain score (*p* < 0.001), bowel function domain score (*p* = 0.024) and prolapse symptoms domain score (*p* = 0.039). Reduced (i.e., weak) anal sphincter contractility during the physical examination was associated with a higher total questionnaire score (*p* = 0.02, Fig. 3b), bladder function domain score (*p* = 0.033), and bowel function domain score (*p* = 0.003). A higher clinical POP-Q Score was associated with a higher prolapse symptoms domain score (*p* = 0.005), but not with total questionnaire score (*p* = 0.17), bladder function domain score (*p* = 0.30), bowel function domain score (*p* = 0.51), or sexual function domain score (*p* = 0.63). There was no significant correlation between the modified Oxford Score (as an ordinal-scaled measure of pelvic floor contractibility) and either the four domain scores (all *p* > 0.40) or the total score (*p* = 0.74) of the pelvic floor questionnaire.

## Discussion

To our knowledge, this is the first study examining pelvic floor function after third degree perineal injury and its subsequent influence on quality of life using a validated questionnaire and physical examination. Additionally, we examined obstetric factors and their effects on long-term pelvic floor function after OASIS. The rate of OASIS following vaginal delivery during our study period was 2.6% for grade III tears and 0.1% for grade IV tears. The literature cites a rate of 1–18%, although the rate of occult injuries is estimated to be around 35%, substantially higher than the reported injury rates [30, 35]. The participation rate of 20% (71 women) was concordant with other studies involving physical examination [25]. Studies using questionnaires alone had a substantially higher participation rate with a return rate of 32–77% [3, 8, 9, 30]. Overall, few studies examine the long-term effect of anal sphincter injury on pelvic floor function. Often these studies focused on stool function, and less on other pelvic floor disorders such as urinary incontinence, prolapse and sexuality. The data and results of these studies differ substantially. A randomized study with an 18-year follow-up reported that women with an OASIS had a higher rate of stool incontinence than women with intact sphincter after delivery. The perineal injury had no influence on urinary incontinence, sexuality or the woman’s well-being [23]. In contrast, other studies reported that women with OASIS had an increased rate of urinary incontinence [9, 28] and experienced a negative effect on sexuality [4, 33].

### Risk factors for pelvic floor insufficiency

Maternal age, BMI, parity, child position, delivery weight and fetal head circumference have been reported as risk factors for pelvic insufficiency following delivery. However, we found no association between these factors and subsequent pelvic function. Similar results are reported by Baud et al. [3]. It is possible that a larger sample size is needed to determine the relevance of these individual factors. Supine maternal delivery position in our collective was associated with a higher bladder function domain score, bowel function domain score, sexual function domain score and total questionnaire score. The literature is inconsistent with regard to supine delivery position and subsequent risks, but a Cochrane analysis from 2017 found no association between delivery position, perineal injury and subsequent pelvic floor function [16]. Similar to the results published in two other studies, vacuum delivery in our study was associated with negative effects on bowel function [3, 7]. Given that vaginal operative delivery is a known risk factor for OASIS, which in turn can negatively affect stool function and thus quality of life, the vaginal operative delivery should be well considered and restricted for indicated cases. Interestingly we found a significant association between epidural anesthesia and higher sexual function domain score and total questionnaire score, indicating a possible long-term negative impact of epidural anesthesia for vaginal delivery on pelvic floor function. This result was surprising, as the current literature reports no documented negative associations between pelvic floor function and epidural anesthesia [12].

### Bladder function and urinary incontinence

The finding of increased SI in patients after OASIS was reported not only in our study but also in other published studies assessing patients after OASIS [3, 23]. Here, 74.6% (53 women) reported symptoms of SI, and 10% (7 women) had daily loss of urine with physical activity. This prevalence was higher than other published results, with rates of stress incontinence reported in the range of 14–21.3% after OASIS [9, 28]. These numbers might be underestimations, because the prevalence of stress incontinence even within the general population is an estimated 48% with ranges from 29 to 75% [21, 36]. The rate of OAB as well as the rate of urge incontinence in this study is in accordance with other studies, reporting percentages of 14–16% OAB in long-term follow-up after delivery with OASIS [9, 23]. Estimates for the rate of OAB in the general population range between 11.5 and 35.6% [6]. Baud et al. compared women with and without OASIS and found an increased incidence of overactive bladder in patients with OASIS [3].

In summary, we found a higher rate of SI following OASIS in comparison to other studies. However, our results have to be interpreted with care, and a causal relationship cannot be inferred based on the association found in our explorative study. Generally speaking, the influence of OASIS on SI and OAB is not clear, as the prevalence in the general population is high and there are a variety of other factors like overweight, increasing age and parity that contribute to the development of these disorders [20]. Large epidemiological studies would be necessary to determine causation.

### Bowel function and stool incontinence

Pregnancy and delivery are known risk factors for the development of stool incontinence. A prospective study showed that in young nulliparous women the rate of flatus incontinence was 12.1% before delivery and rose to 28.3% 6 months after delivery [15]. Multiple studies have shown that injury to the anal sphincter significantly increased the risk of stool incontinence [3, 7, 9, 34]. The reported rate of flatus incontinence and liquid stool incontinence after vaginal delivery without sphincter injury was estimated as 6–37% and 2–9%, respectively [3, 7, 30]. Following cesarean delivery, the rates of flatus incontinence and liquid stool incontinence reported were 10–15% and 4–7%, respectively [7, 25]. The reported rate of stool incontinence after OASIS varies widely in the literature, ranging from 15–61% [25, 27, 29]. In our study, the rate of frequent flatus incontinence was almost 30% (46 women); soft stool incontinence and formed stool incontinence were reported by 18.3% (13 women) and 1.4% (1 woman), respectively. Similar percentages were reported in the study from Evers et al., in which 90 women were interviewed and specifically asked about symptoms 5–10 years following OASIS [7]. Another study reported a considerably higher rate of flatus and stool incontinence 6 years after delivery with anal sphincter tear with a flatus incontinence rate over 50% and a formed stool incontinence rate of 5.9% [3].

### Genital prolapse

To our knowledge, there are no documented studies examining the influence of OASIS on genital prolapse. It is widely accepted that an increased number of vaginal deliveries increases the risk of prolapse. The data examining the influence of vaginal operative delivery or episiotomy are inconsistent [18, 32]. In our cohort, we found no correlation between vaginal operative delivery or episiotomy and the presence of prolapse or prolapse complaints (including pain or pressure). The rates of 31.0% grade 1 (22 women) and 18.3% grade 2 (13 women) pelvic organ prolapse as found in our study are higher than what is reported in the general population. A Swedish study of women between ages 20–59 reported a prevalence of any degree of prolapse of 30.8% [26]. The higher rate of pelvic organ prolapse observed in our study may be a result of the complexity of the delivery, as evidenced by the OASIS, and the overall increased strain on the pelvic floor, as reflected by the positive correlation between duration of the first (latent) stage of labor (min) and prolapse symptoms domain score.

### Sexuality

More than a third of the women (36.6%–26 women) in this study reported no or seldom sexual activity, with reasons stated being–among others–dyspareunia, decreased interest or decreased lubrication. A long-term (18 years postpartum) follow-up study revealed no significant difference with regard to sexual symptoms between women with and without OASIS [23]. In contrast, Baud et al. showed that women 6 years after OASIS reported more problems with lubrication, ability to orgasm, and more pain in comparison to women without sphincter injury [3]. The effect of pregnancy and delivery including the role as parent on sexuality and sexual function is extremely complex [17, 36]. Thus, single factors such as an OASIS event probably cannot be used to reliably predict sexual functioning in the long term, and larger multivariable prospective and epidemiological studies are needed to elucidate independent effects of various birth-related factors on subsequent sexual function.

In summary, we found a high rate of pelvic floor dysfunction 2.4–11.1 years after an OASIS event during vaginal birth with a urinary stress incontinence rate of almost 75%, a genital prolapse rate of about 50%, a flatus incontinence rate of about 65% and a stool incontinence rate of about 18%. In spite of these high percentages, only few women reported that they were moderately or even greatly bothered by these problems (about 10% for bladder, bowel and sexual function and about 3% for prolapse symptoms).

A limitation of this study is that there is no control cohort of women with vaginal delivery without sphincter injury or with cesarean section to assess the influence of OASIS on the outcome. Additionally, this study, similar to most other studies addressing the issue of long-term pelvic floor disorders after OASIS, has no clinical examination before or during the pregnancy. As a result, we could not directly assess the pregnancy or delivery associated changes. Moreover, OASIS were not consequently sub-classified into A, B and C, which is known to be critical for long-term function. Finally, our study cohort may be biased, as women with pelvic floor associated complaints or symptoms could be more likely to participate in a study with clinical examination than women without such complaints. A strength of this study is the relatively high number of women who participated years after the delivery. Additionally, this study comprised not only a validated questionnaire that recorded both symptoms and effects on quality of life, but also a clinical examination to assess pelvic floor disorders.

This study showed that the results of a simple and easily feasible clinical examination comprising the cough test, assessment of the anal and pelvic floor sphincter contractibility, and the evaluation of the pelvic floor prolapse were significantly associated with long-term self-reported pelvic floor complaints following vaginal delivery with an OASIS event. About 10% of women stated to have an impairment due to pelvic floor dysfunction. Importantly, none of the women had talked to a physician in the years after delivery, except those who had the appointment 8 to 12 weeks after delivery. This indicates that pelvic floor disorders still represent a taboo topic. In the literature, it is documented that only an estimated 15% of women with stool incontinence discuss the problem with their physician [14], and only about 15% of polled women spoke about their sexual problems after delivery [2, 17].

## Conclusion

The rate of pelvic floor disorders following OASIS is high and can be detected by simple clinical examination, but many women regard this issue still as a taboo topic and do not dare to speak about it. Therefore, the health-care professionals should actively address the problem of pelvic floor disorders following an OASIS event during birth and offer therapy options for the women with persisting or especially incurring complaints in the long-term follow-up. The treating physician is responsible for the active discussion to provide treatment and further diagnostic tests for the estimated 10% of women who suffer from a decreased quality of life because of these disorders.
